# (11a*S*)-8-Hydr­oxy-7-meth­oxy-2,3,5,10,11,11a-hexa­hydro-1*H*-pyrrolo[2,1-*c*][1,4]benzodiazepine-3,11-dione

**DOI:** 10.1107/S1600536807066056

**Published:** 2007-12-12

**Authors:** Dong-Mei Zhao, Chao Ma, Yu Sha, Jing-Hong Liu, Mao-Sheng Cheng

**Affiliations:** a School of Pharmaceutical Engineering, Shenyang Pharmaceutical University, Mail Box 40, 103 Wenhua Road, Shenhe District, Shenyang 110016, People’s Republic of China

## Abstract

The title chiral compound, C_13_H_14_N_2_O_4_, was prepared by an intra­cyclization reaction of methyl (*S*)-1-(4-hydr­oxy-5-meth­oxy-2-nitro­benz­yl)-5-oxopyrrolidine-2-carboxyl­ate in the presence of ethanol and iron. The five-membered substituted pyrrole ring adopts an approximate envelope conformation, while the seven-membered substituted diazepine ring displays a twist-boat conformation. Inter­molecular O—H⋯O and N—H⋯O hydrogen bonding helps to stabilize the crystal structure.

## Related literature

For general background, see: Bose *et al.* (1992 ▶); Hu *et al.* (2001 ▶); Kamal *et al.* (2002 ▶); Thurston & Bose (1994 ▶). For a related structure, see: Cheng *et al.* (2007 ▶).
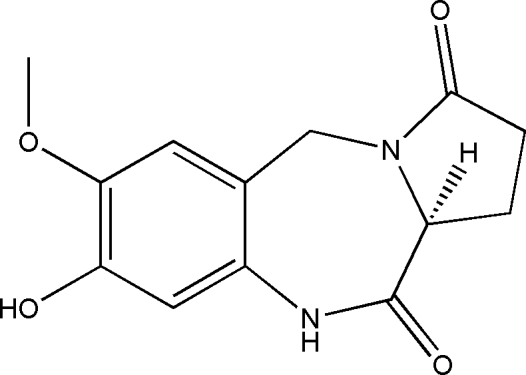


## Experimental

### 

#### Crystal data

C_13_H_14_N_2_O_4_
*M* *_r_* = 262.26
Monoclinic, 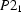

*a* = 6.3819 (7) Å
*b* = 9.3139 (10) Å
*c* = 10.3673 (11) Å
β = 103.621 (1)°
*V* = 598.90 (11) Å^3^
*Z* = 2
Mo *K*α radiation
μ = 0.11 mm^−1^
*T* = 187 (2) K
0.48 × 0.26 × 0.15 mm

#### Data collection

Bruker SMART APEX CCD area-detector diffractometer
Absorption correction: none
3169 measured reflections
1168 independent reflections
1149 reflections with *I* > 2σ(*I*)
*R*_int_ = 0.013

#### Refinement

*R*[*F* ^2^ > 2σ(*F* ^2^)] = 0.030
*wR*(*F* ^2^) = 0.074
*S* = 1.12
1168 reflections
174 parameters
1 restraint
H-atom parameters constrained
Δρ_max_ = 0.14 e Å^−3^
Δρ_min_ = −0.19 e Å^−3^

### 

Data collection: *SMART* (Bruker, 1997 ▶); cell refinement: *SAINT* (Bruker, 1999 ▶); data reduction: *SAINT*; program(s) used to solve structure: *SHELXS97* (Sheldrick, 1997 ▶); program(s) used to refine structure: *SHELXL97* (Sheldrick, 1997 ▶); molecular graphics: *SHELXTL-Plus* (Siemens, 1990 ▶); software used to prepare material for publication: *SHELXL97*.

## Supplementary Material

Crystal structure: contains datablocks I, global. DOI: 10.1107/S1600536807066056/xu2391sup1.cif

Structure factors: contains datablocks I. DOI: 10.1107/S1600536807066056/xu2391Isup2.hkl

Additional supplementary materials:  crystallographic information; 3D view; checkCIF report

## Figures and Tables

**Table 1:** Hydrogen-bond geometry (Å, °)

*D*—H⋯*A*	*D*—H	H⋯*A*	*D*⋯*A*	*D*—H⋯*A*
O1—H1⋯O4^i^	0.84	1.95	2.707 (2)	150
N1—H1*A*⋯O1^ii^	0.97	2.11	3.023 (2)	157

Symmetry codes: (i) 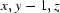; (ii) 
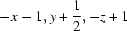
.

**Fig. 1.**
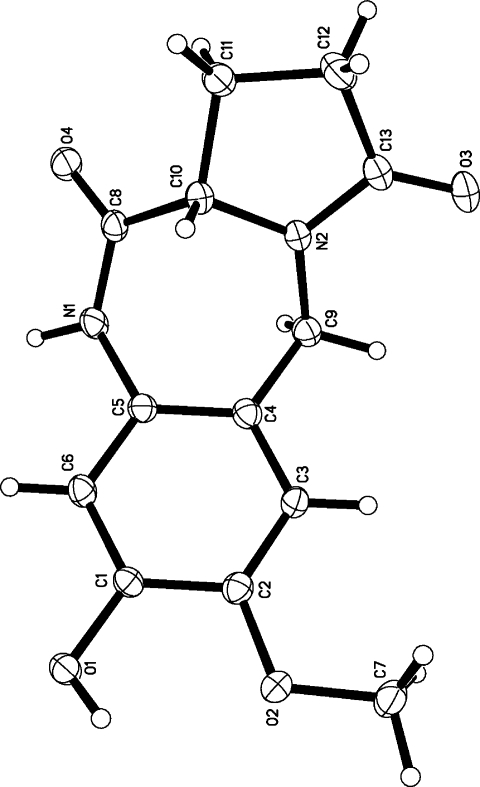
The structure of the title compound, showing 30% probability displacement ellipsoids and the atom-numbering scheme.

## supplementary crystallographic information

### Comment

Pyrrolo[2,1-*c*][1,4]benzodiazepines (PBDs) are a group of potent, naturally occurring antitumor antibiotics produced by Streptomyces species (Kamal *et al.*, 2002; Thurston & Bose, 1994). Naturally occurring Pyrrolo[2,1-*c*][1,4]benzodiazepines (PBDs) have attracted the attention of many researchers largely because of the potent anticancer activity exhibited in most of the compounds with this ring system (Hu *et al.*, 2001; Bose *et al.*, 1992). As PBDs compounds are of great pharmaceutical importance, we determined the title chiral compound's crystal structure. The molecular is shown in Fig. 1 and the bond lengths and angles are within normal ranges. PBD ring involes in a twisted conformation, similar to a related structure (Cheng *et al.*, 2007). The seven-membered ring C5—C4—C9—N2—C10—C8—N1 (substituted diazepine) is far from planar, and its shape approximates to a twist boat. In this description applied to the title compound (Fig. 1), atoms C8, C9, N1 and N2 form the bottom of the boat (deviation from the mean N1/C9/N2/C8 plane = 0.125 (2) Å), C10 the prow, and C4 and C5 the stern [deviations from the C8/C9/N1/N2 mean plane = 0.567, 0.885, 0.948 Å, respectively]. The bond length of the carbonyl groups C8=O4 and C13=O3 of 1.228 (2) and 1.225 (3) Å, respectively, are somewhat longer than typical carbonyl bonds. This may be due to the fact that atoms O3 and O4 participate in intermolecular van der Waals forces. The five-membered ring N2—C10—C11—C12—C13 (substituted pyrrole) is non-planar and adopts nearly envelope conformation (deviation from the mean C10/N2/C13/C12 plane = 0.021 (5) Å). The C11 atom is located above the plane [deviations from the C10/N2/C13/C12 mean plane = 0.449 Å]. Atom C10 of the title molecule is chiral: S configuration was assigned to this atom based on the known chirality of the equivalent atom in the starting material. In the crystal structure, intermolecular O—H···O and N—H···O hydrogen bonds link the molecules together (Table 1) and help to stabilize the structure.

### Experimental

(*S*)-1-(4-Hydroxy-5-mythoxy-2-nitrobenzyl)-5-oxopyrrolidine-2-carboxylic acid methyl ester (8.10 g, 25 mmol) was dissolved in ethanol (150 ml). Fe (3.36 g, 60 mmol) was added and the solution was heated to reflux for 30 min. The mixture was filtered and the filtrate was concentrated under vacuum. The pure product was obtained through silica gel chromatography (eluant: petroleum ether/ethyl acetate, 2:1). Single crystals suitable for X-ray diffraction were obtained by slow evaporation of a dilute solution of the title compound in ethyl acetate at room temperature.

### Refinement

All H atoms were placed in geometrically idealized positions and constrained to ride on their parent atoms, with N—H = 0.97 Å, O—H = 0.84 Å, and C—H = 0.95, 0.99, 0.98 and 1.00 Å for phenyl, methylene, methyl and tertiary H atoms, respectively, with *U*_iso_(H) = *xU*_eq_(C,*N*,*O*), where *x* = 1.5 for methyl H and hydroxyl H, and *x* = 1.2 for all other H atoms. Based on known chirality of the equivalent atom in the starting material, the S chirality at C10 was assigned. Friedels pairs were merged.

### Crystal data

**Table:**

C_13_H_14_N_2_O_4_	*F*_000_ = 276
*M**_r_* = 262.26	*D*_x_ = 1.454 Mg m^−^^3^
Monoclinic, *P*2_1_	Mo *K*α radiation λ = 0.71073 Å
Hall symbol: P 2yb	Cell parameters from 2332 reflections
*a* = 6.3819 (7) Å	θ = 3.0–25.3º
*b* = 9.3139 (10) Å	µ = 0.11 mm^−^^1^
*c* = 10.3673 (11) Å	*T* = 187 (2) K
β = 103.621 (1)º	Block, colorless
*V* = 598.90 (11) Å^3^	0.48 × 0.26 × 0.15 mm
*Z* = 2	

### Data collection

**Table:**

Bruker SMART APEX CCD area-detector diffractometer	1149 reflections with *I* > 2σ(*I*)
Radiation source: fine-focus sealed tube	*R*_int_ = 0.013
Monochromator: graphite	θ_max_ = 25.3º
*T* = 187(2) K	θ_min_ = 3.0º
φ and ω scans	*h* = −7→7
Absorption correction: none	*k* = −5→11
3169 measured reflections	*l* = −12→11
1168 independent reflections	

### Refinement

**Table:**

Refinement on *F*^2^	Secondary atom site location: difference Fourier map
Least-squares matrix: full	Hydrogen site location: inferred from neighbouring sites
*R*[*F*^2^ > 2σ(*F*^2^)] = 0.030	H-atom parameters constrained
*wR*(*F*^2^) = 0.074	*w* = 1/[σ^2^(*F*_o_^2^) + (0.0425*P*)^2^ + 0.0991*P*] where *P* = (*F*_o_^2^ + 2*F*_c_^2^)/3
*S* = 1.12	(Δ/σ)_max_ = 0.004
1168 reflections	Δρ_max_ = 0.14 e Å^−^^3^
174 parameters	Δρ_min_ = −0.19 e Å^−^^3^
1 restraint	Extinction correction: none
Primary atom site location: structure-invariant direct methods	

### Special details

**Table:**

Geometry. All e.s.d.'s (except the e.s.d. in the dihedral angle between two l.s. planes) are estimated using the full covariance matrix. The cell e.s.d.'s are taken into account individually in the estimation of e.s.d.'s in distances, angles and torsion angles; correlations between e.s.d.'s in cell parameters are only used when they are defined by crystal symmetry. An approximate (isotropic) treatment of cell e.s.d.'s is used for estimating e.s.d.'s involving l.s. planes.
Refinement. Refinement of *F*^2^ against ALL reflections. The weighted *R*-factor *wR* and goodness of fit *S* are based on *F*^2^, conventional *R*-factors *R* are based on *F*, with *F* set to zero for negative *F*^2^. The threshold expression of *F*^2^ > σ(*F*^2^) is used only for calculating *R*-factors(gt) *etc*. and is not relevant to the choice of reflections for refinement. *R*-factors based on *F*^2^ are statistically about twice as large as those based on *F*, and *R*- factors based on ALL data will be even larger.

### Fractional atomic coordinates and isotropic or equivalent isotropic displacement parameters (Å^2^)

**Table:**

	*x*	*y*	*z*	*U*_iso_*/*U*_eq_	
C1	−0.1968 (3)	0.3136 (2)	0.6150 (2)	0.0254 (4)	
C2	0.0218 (3)	0.2900 (2)	0.6730 (2)	0.0283 (5)	
C3	0.1550 (3)	0.4061 (3)	0.7152 (2)	0.0290 (5)	
H3	0.3026	0.3905	0.7566	0.035*	
C4	0.0751 (3)	0.5463 (3)	0.6978 (2)	0.0275 (5)	
C5	−0.1437 (3)	0.5676 (2)	0.6429 (2)	0.0263 (4)	
C6	−0.2799 (4)	0.4511 (3)	0.6011 (2)	0.0280 (5)	
H6	−0.4289	0.4662	0.5632	0.034*	
C7	0.3005 (4)	0.1165 (3)	0.7439 (3)	0.0375 (5)	
H7A	0.3303	0.1447	0.8375	0.056*	
H7B	0.3962	0.1695	0.6995	0.056*	
H7C	0.3253	0.0132	0.7374	0.056*	
C8	−0.1959 (3)	0.8199 (2)	0.7104 (2)	0.0281 (5)	
C9	0.2252 (3)	0.6725 (2)	0.7329 (2)	0.0304 (5)	
H9A	0.3751	0.6369	0.7620	0.037*	
H9B	0.2159	0.7322	0.6528	0.037*	
C10	−0.0465 (3)	0.7914 (3)	0.8459 (2)	0.0280 (5)	
H10	−0.1028	0.7076	0.8878	0.034*	
C11	−0.0166 (3)	0.9189 (3)	0.9420 (2)	0.0360 (5)	
H11A	−0.1253	0.9172	0.9962	0.043*	
H11B	−0.0277	1.0112	0.8935	0.043*	
C12	0.2116 (4)	0.8965 (3)	1.0287 (2)	0.0402 (6)	
H12A	0.2842	0.9897	1.0540	0.048*	
H12B	0.2073	0.8425	1.1103	0.048*	
C13	0.3266 (3)	0.8111 (3)	0.9419 (2)	0.0310 (5)	
N1	−0.2336 (3)	0.7085 (2)	0.62395 (18)	0.0314 (4)	
H1A	−0.3508	0.7192	0.5458	0.038*	
N2	0.1759 (3)	0.76080 (19)	0.83733 (17)	0.0267 (4)	
O1	−0.3329 (2)	0.20085 (17)	0.57207 (15)	0.0316 (4)	
H1	−0.2729	0.1238	0.6029	0.047*	
O2	0.0812 (2)	0.14895 (17)	0.68141 (17)	0.0352 (4)	
O3	0.5206 (2)	0.7896 (2)	0.96020 (16)	0.0416 (4)	
O4	−0.2877 (3)	0.93534 (18)	0.68144 (17)	0.0373 (4)	

### Atomic displacement parameters (Å^2^)

**Table:**

	*U*^11^	*U*^22^	*U*^33^	*U*^12^	*U*^13^	*U*^23^
C1	0.0268 (10)	0.0274 (11)	0.0211 (9)	−0.0026 (9)	0.0038 (8)	−0.0001 (9)
C2	0.0280 (11)	0.0297 (11)	0.0279 (11)	0.0021 (9)	0.0078 (8)	0.0001 (9)
C3	0.0229 (10)	0.0323 (12)	0.0309 (11)	0.0032 (9)	0.0047 (8)	−0.0027 (10)
C4	0.0264 (10)	0.0303 (11)	0.0253 (10)	−0.0007 (9)	0.0055 (8)	−0.0016 (9)
C5	0.0284 (10)	0.0255 (11)	0.0228 (9)	0.0015 (9)	0.0017 (8)	0.0005 (9)
C6	0.0255 (10)	0.0311 (12)	0.0247 (10)	−0.0005 (9)	0.0003 (8)	0.0023 (9)
C7	0.0272 (11)	0.0336 (12)	0.0514 (14)	0.0057 (9)	0.0092 (9)	0.0039 (11)
C8	0.0219 (9)	0.0255 (11)	0.0353 (11)	−0.0025 (9)	0.0036 (8)	0.0026 (10)
C9	0.0271 (10)	0.0322 (12)	0.0320 (11)	−0.0021 (9)	0.0070 (9)	−0.0034 (9)
C10	0.0236 (10)	0.0306 (11)	0.0296 (11)	−0.0027 (9)	0.0059 (8)	0.0006 (9)
C11	0.0305 (11)	0.0423 (14)	0.0357 (11)	−0.0042 (11)	0.0086 (9)	−0.0097 (11)
C12	0.0354 (12)	0.0521 (16)	0.0309 (11)	−0.0078 (11)	0.0035 (9)	−0.0089 (11)
C13	0.0272 (10)	0.0333 (12)	0.0295 (10)	−0.0055 (9)	0.0010 (8)	0.0034 (10)
N1	0.0294 (9)	0.0277 (10)	0.0304 (9)	0.0014 (9)	−0.0066 (7)	0.0029 (9)
N2	0.0216 (8)	0.0298 (10)	0.0273 (9)	−0.0013 (7)	0.0027 (7)	−0.0013 (7)
O1	0.0300 (8)	0.0249 (8)	0.0350 (8)	−0.0020 (7)	−0.0019 (6)	0.0021 (7)
O2	0.0277 (8)	0.0272 (9)	0.0477 (10)	0.0039 (7)	0.0029 (7)	−0.0019 (7)
O3	0.0246 (8)	0.0513 (11)	0.0437 (10)	−0.0034 (8)	−0.0021 (7)	−0.0010 (9)
O4	0.0328 (9)	0.0254 (8)	0.0482 (9)	0.0019 (7)	−0.0013 (7)	0.0036 (7)

### Geometric parameters (Å, °)

**Table:**

C1—O1	1.369 (3)	C8—C10	1.524 (3)
C1—C6	1.381 (3)	C9—N2	1.452 (3)
C1—C2	1.400 (3)	C9—H9A	0.9900
C2—O2	1.364 (3)	C9—H9B	0.9900
C2—C3	1.382 (3)	C10—N2	1.471 (3)
C3—C4	1.398 (3)	C10—C11	1.532 (3)
C3—H3	0.9500	C10—H10	1.0000
C4—C5	1.392 (3)	C11—C12	1.535 (3)
C4—C9	1.506 (3)	C11—H11A	0.9900
C5—C6	1.394 (3)	C11—H11B	0.9900
C5—N1	1.427 (3)	C12—C13	1.514 (3)
C6—H6	0.9500	C12—H12A	0.9900
C7—O2	1.429 (3)	C12—H12B	0.9900
C7—H7A	0.9800	C13—O3	1.224 (3)
C7—H7B	0.9800	C13—N2	1.353 (3)
C7—H7C	0.9800	N1—H1A	0.9699
C8—O4	1.228 (3)	O1—H1	0.8400
C8—N1	1.355 (3)		
			
O1—C1—C6	118.58 (18)	C4—C9—H9B	109.1
O1—C1—C2	120.7 (2)	H9A—C9—H9B	107.8
C6—C1—C2	120.7 (2)	N2—C10—C8	112.39 (17)
O2—C2—C3	126.32 (19)	N2—C10—C11	102.46 (16)
O2—C2—C1	114.37 (19)	C8—C10—C11	114.80 (19)
C3—C2—C1	119.3 (2)	N2—C10—H10	109.0
C2—C3—C4	120.79 (18)	C8—C10—H10	109.0
C2—C3—H3	119.6	C11—C10—H10	109.0
C4—C3—H3	119.6	C10—C11—C12	103.35 (19)
C5—C4—C3	119.1 (2)	C10—C11—H11A	111.1
C5—C4—C9	120.5 (2)	C12—C11—H11A	111.1
C3—C4—C9	120.41 (19)	C10—C11—H11B	111.1
C6—C5—C4	120.5 (2)	C12—C11—H11B	111.1
C6—C5—N1	118.15 (18)	H11A—C11—H11B	109.1
C4—C5—N1	121.28 (19)	C13—C12—C11	104.43 (18)
C1—C6—C5	119.54 (19)	C13—C12—H12A	110.9
C1—C6—H6	120.2	C11—C12—H12A	110.9
C5—C6—H6	120.2	C13—C12—H12B	110.9
O2—C7—H7A	109.5	C11—C12—H12B	110.9
O2—C7—H7B	109.5	H12A—C12—H12B	108.9
H7A—C7—H7B	109.5	O3—C13—N2	124.7 (2)
O2—C7—H7C	109.5	O3—C13—C12	127.5 (2)
H7A—C7—H7C	109.5	N2—C13—C12	107.80 (18)
H7B—C7—H7C	109.5	C8—N1—C5	127.59 (17)
O4—C8—N1	120.58 (19)	C8—N1—H1A	117.1
O4—C8—C10	122.5 (2)	C5—N1—H1A	114.5
N1—C8—C10	116.85 (19)	C13—N2—C9	123.76 (18)
N2—C9—C4	112.66 (17)	C13—N2—C10	113.48 (18)
N2—C9—H9A	109.1	C9—N2—C10	122.48 (16)
C4—C9—H9A	109.1	C1—O1—H1	109.5
N2—C9—H9B	109.1	C2—O2—C7	117.41 (18)

### Hydrogen-bond geometry (Å, °)

**Table:**

*D*—H···*A*	*D*—H	H···*A*	*D*···*A*	*D*—H···*A*
O1—H1···O4^i^	0.84	1.95	2.707 (2)	150
N1—H1A···O1^ii^	0.97	2.11	3.023 (2)	157.2

Symmetry codes: (i) *x*, *y*−1, *z*; (ii) −*x*−1, *y*+1/2, −*z*+1.
